# Muscle Mitochondrial Respiration and Cardiorespiratory Fitness Contribute to Slower Walking Speed of Older Individuals Who Identify as Black

**DOI:** 10.1111/acel.70040

**Published:** 2025-03-27

**Authors:** Paul M. Coen, Fanchao Yi, Li‐Yung Lui, Giovanna Distefano, Sofhia V. Ramos, Bret H. Goodpaster, Peggy M. Cawthon, Russell T. Hepple, Stephen B. Kritchevsky, Steven R. Cummings, Gregory J. Tranah, Anne B. Newman, James P. DeLany

**Affiliations:** ^1^ Translational Research Institute AdventHealth Orlando Florida USA; ^2^ California Pacific Medical Center Research Institute San Francisco California USA; ^3^ University of California San Francisco San Francisco California USA; ^4^ Department of Physical Therapy University of Florida Gainesville Florida USA; ^5^ Department of Internal Medicine Wake Forest University School of Medicine Winston‐Salem North Carolina USA; ^6^ Department of Epidemiology School of Public Health, University of Pittsburgh Pittsburgh Pennsylvania USA

**Keywords:** aging, mitochondria, skeletal muscle

## Abstract

In the United States, older adults who self‐identify as Black have a disproportionately higher incidence of mobility disability compared to those who are White. Whether older adults who are Black also have lower fitness and mitochondrial energetics has not been adequately investigated. The study of muscle, mobility and aging (SOMMA) examined 879 participants aged ≥ 70 years old, including 116 who self‐identified as Black. Mitochondrial respiration (Max OXPHOS) was measured in permeabilized fibers from muscle biopsies. Cardiorespiratory fitness (VO_2_ peak) was determined by a cardiopulmonary exercise test. Education, income, financial resources, race, sex, and age were determined by self‐report. We used propensity score matching to match Blacks with Whites with a 1:1 ratio. Black (*n* = 90) and White (*n* = 90) groups were matched for age, sex, SOMMA multimorbidity index, BMI, muscle mass, physical activity, marital status, educational achievement, and whether financial needs were met (all *p* > 0.05). Despite being well matched for these variables, those who identified as Black had a slower 400‐m walking speed (0.97 vs. 1.03 m/s, *p* = 0.014), lower Max OXPHOS (50.8 vs. 60.9 (pmol/(s*mg)), *p* = 0.0002), and lower cardiorespiratory fitness (1391 vs. 1566 mL/min, *p* = 0.007) when compared to those who identified as White. Multivariate regression showed that VO_2_ peak and Max OXPHOS, but not socioeconomic factors, attenuated the race difference in 400‐m walking speed. In conclusion, while the etiology of race differences in mobility is multifactorial, our data indicate that muscle mitochondrial respiration and cardiorespiratory fitness may contribute to the slower walking speed of individuals who identify as Black compared to White.

## Graphical abstract

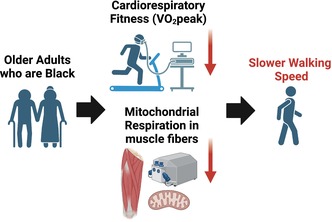

## 1 Introduction

Age‐associated loss of mobility is a serious healthcare issue for the aging US population. Older adults who identify as non‐Hispanic Black have a disproportionately higher incidence and prevalence of mobility disability (Okoro et al. 2018; Thorpe Jr. et al. 2011). Large cohort studies, including the Health ABC Study, have also reported racial differences in the physical function of older adults (Courtney‐Long et al. 2015; Thorpe Jr. et al. 2011). While both biological and social factors likely contribute to the development of health disparities, the relative contribution of each is difficult to evaluate.

Socioeconomic status (SES) encompassing educational attainment, income, and financial resources, is strongly related to physical function in late life, with the economically or educationally disadvantaged consistently showing poorer function (8,9) (Coppin et al. 2006). In the US population, race and SES are highly correlated, with Blacks having markedly fewer socioeconomic resources than Whites (Braveman et al. 2005; LaVeist 2005; Thorpe Jr., Brandon, and LaVeist 2008). The strong interrelationship between race and SES complicates efforts to disentangle the relative associations of each with functional impairment. Although observed race‐related differences in physical function diminish after accounting for education and income (Guralnik et al. 1993; Schoenbaum and Waidmann 1997), a residual association remains. For example, in a very large study of 3,429,523 individuals, a higher prevalence of disability was observed in non‐Latino Blacks compared to non‐Latino Whites for those with both low and high education (SES measure) (Siordia 2015). Lower SES was associated with a greater prevalence of disability, but lower SES status alone did not explain greater mobility disability limitations in non‐Latino Black adults compared to non‐Latino White adults, indicating that other factors may be involved in the greater disability of non‐Latino Black adults. It should be noted, however, that many other social factors could contribute to health disparities across race, and unexplained differences may be due to imperfect data and the mismatch between a lifetime of exposure and measures of current SES status.

While the physiological etiology of mobility loss is complex, an appreciation for skeletal muscle mitochondrial function and cardiorespiratory fitness as key factors has emerged (Schrack et al. 2010). Our group and others have recently contributed to this paradigm by demonstrating that mitochondrial respiration is a key property of skeletal muscle that, along with cardiorespiratory fitness, likely supports walking speed and physical function in older adults (Coen et al. 2013; Gonzalez‐Freire et al. 2018). In addition, we recently showed that skeletal muscle mitochondrial respiration is lower in young women who are Black compared to those who are White and is linked with lower cardiorespiratory fitness and lower resting metabolic rate (RMR) (DeLany et al. 2014; Toledo et al. 2018). Others have shown that young Black women have lower cardiorespiratory fitness (VO_2_ peak) and that differences in hemoglobin levels and skeletal muscle oxidative capacity were shown to be associated with, but did not fully explain, the racial difference (Hunter et al. 2000; Hunter et al. 2001). Older Black adults have greater strength than older White adults but have lower specific strength, reflecting a lower amount of strength relative to lean body mass (Goodpaster et al. 2006; Newman et al. 2003). As such, in addition to SES factors, differences in fitness, strength, and mitochondrial energetics could contribute to racial differences in mobility in older adults.

The present analysis leveraged data obtained from the study of muscle, mobility and aging (SOMMA) to investigate the relative associations of SES factors, including education and income, along with skeletal muscle energetics and cardiorespiratory fitness to explain race differences in mobility in a large cohort of older adults.

## 2 Results

### 2.1 Characteristics of Participants Who Self‐Identify as Black or White in SOMMA

A total of 879 participants provided consent and completed baseline measurements across both clinical sites. The number of SOMMA participants who self‐identified as White was *n* = 745, and those who self‐identified as Black was *n* = 116. There were limited numbers of participants in other self‐reported ethnicity/race categories, so our analysis focused on those who self‐identified as White or Black only (Cummings et al. 2023). The ratio of White to Black participants was similar at both clinical sites. In unadjusted group comparisons, White participants were slightly older and were more likely to be female. SOMMA participants who were Black had a higher BMI, greater D3CR muscle mass and lower physical activity, and a greater number of chronic conditions (Table 1). With respect to differences in socioeconomic factors, SOMMA participants who were Black were more likely to be divorced, had a lower educational achievement, and generally had lower income and financial resources. Black participants also had greater exposure to adverse childhood experiences (Table 1 and Table S1).

**TABLE 1:** Race differences in clinical and socioeconomic characteristics.

Variable		Before PS matching			After PS matching		
		White (*n* = 745)	Black (*n* = 116)	*p*	White (*n* = 90)	Black (*n* = 90)	*p*
Clinical site, Pittsburgh		374 (50.2)	58 (50.0)	1.000	44 (48.9)	44 (48.9)	1.000
*Participant sex, male		314 (42.1)	33 (28.4)	**0.006**	31 (34.4)	26 (28.9)	0.522
*Age, years		76.57+/−0.183	75.13+/−0.476	**0.004**	74.86+/−0.422	75.09+/−0.561	0.740
*BMI, kg/m^2^		27.30+/−0.164	29.73+/−0.421	**< 0.001**	29.24+/−0.477	29.56+/−0.490	0.643
*D3CR muscle mass, kg		21.81+/−0.244	23.54+/−0.631	**0.012**	24.17+/−0.770	23.70+/−0.673	0.643
*CHAMPS: Strength exercises and all walking, hours/week		5.848+/−0.203	4.119+/−0.362	**< 0.001**	3.919+/−0.415	4.150+/−0.423	0.698
Mean total daily step count, steps		6974+/−125	5608+/−265	**< 0.001**	6205+/−340.7	5804+/−296.0	0.374
*SOMMA multimorbidity index, no. of chronic conditions	0	329 (44.5)	35 (32.1)	**0.028**	32 (35.6)	30 (33.3)	0.734
	1	281 (38.0)	55 (50.5)		37 (41.1)	42 (46.7)	
	2+	129 (17.5)	19 (17.4)		21 (23.3)	18 (20.0)	
*Current marital status	Married	397 (53.6)	33 (28.4)	**< 0.001**	28 (31.1)	29 (32.2)	0.983
	Widowed	169 (22.8)	31 (26.7)		21 (23.3)	23 (25.6)	
	Separated	3 (0.40)	3 (2.6)		2 (2.2)	2 (2.2)	
	Divorced	125 (16.9)	40 (34.5)		29 (32.2)	28 (31.1)	
	Never married	47 (6.3)	9 (7.8)		10 (11.1)	8 (8.9)	
Adverse childhood experience, questionnaire score (0–6)	0	311 (47.8)	33 (37.5)	**0.012**	29 (36.7)	23 (32.9)	0.354
	1	146 (22.5)	18 (20.5)		14 (17.7)	17 (24.3)	
	2	102 (15.7)	13 (14.8)		18 (22.8)	9 (12.9)	
	3	44 (6.8)	10 (11.4)		9 (11.4)	8 (11.4)	
	4	19 (2.9)	9 (10.2)		3 (3.8)	8 (11.4)	
	5	22 (3.4)	3 (3.4)		5 (6.3)	3 (4.3)	
	6	6 (0.9)	2 (2.3)		1 (1.3)	2 (2.9)	
*Education level attained	High school or lower	88 (11.9)	21 (18.6)	**0.002**	12 (13.3)	14 (15.6)	0.904
	Some college	164 (22.2)	35 (31.0)		29 (32.2)	28 (31.1)	
	College graduate	193 (26.1)	26 (23.0)		24 (26.7)	22 (24.4)	
	Postcollege	281 (38.0)	26 (23.0)		24 (26.7)	23 (25.6)	
	Other	13 (1.8)	5 (4.4)		1 (1.1)	3 (3.3)	
Mother employment: What portion of time did your mother work outside the home when you were growing up	None at all	303 (41.2)	21 (19.6)	**< 0.001**	37 (41.1)	15 (17.0)	**< 0.001**
	Some of the time	303 (41.2)	38 (35.5)		33 (36.7)	32 (36.4)	
	All of the time	122 (16.6)	45 (42.1)		20 (22.2)	38 (43.2)	
	Never lived with mother/mother was not alive	7 (0.9)	3 (2.8)		0 (0.00)	3 (3.41)	
Father employment: Before age 16, was there a time of several months or more when your father had no job	No	531 (81.1)	57 (67.1)	**< 0.001**	65 (82.3)	48 (69.6)	**0.019**
	Yes	97 (14.8)	10 (11.8)		10 (12.7)	7 (10.1)	
	Father never worked or always disabled	1 (0.1)	0 (0.0)		0 (0.0)	0 (0.0)	
	Never lived with father or father was not alive	26 (4.0)	18 (21.2)		4 (5.06)	14 (20.3)	
Income level	Less than $25,000	61 (10.2)	20 (25.6)	**< 0.001**	16 (21.1)	18 (26.9)	0.463
	$25,000–$50,000	153 (25.7)	26 (33.3)		28 (36.8)	19 (28.4)	
	$50,000–$75,000	132 (22.1)	19 (24.4)		15 (19.7)	18 (26.9)	
	$75,000–$125,000	150 (25.2)	7 (9.0)		12 (15.8)	6 (9.0)	
	More than $125,000	100 (16.8)	6 (7.7)		5 (6.6)	6 (9.0)	
*How well does the amount of money you (and your partner) have take care of your needs	Poorly	16 (2.2)	3 (2.9)	**< 0.001**	3 (3.3)	2 (2.2)	1.000
	Fairly well	208 (28.7)	59 (56.2)		50 (55.6)	50 (55.6)	
	Very well	502 (69.1)	43 (41.0)		37 (41.1)	38 (42.2)	
Stock or stock mutual funds	No	203 (29.6)	73 (73.0)	**< 0.001**	38 (44.2)	56 (68.3)	**0.002**
	Yes	483 (70.4)	27 (27.0)		48 (55.8)	26 (31.7)	

*Note:* Group comparisons are made for the whole cohort before propensity score matching (White, *n* = 745 and Black, *n* = 116), and in matched groups (White, *n* = 90 and Black, *n* = 90) after propensity score matching. Continuous variables were tested using the two‐sample t‐test, and categorical variables were tested using Fisher's exact test. Continuous variables were presented using mean and standard error (SE) by race, and categorical variables were presented using frequency and proportion by race. *Indicates variables that were used in the propensity score model to match groups. Boldface indicates significance accepted at *p* < 0.05.

**FIGURE 1:**
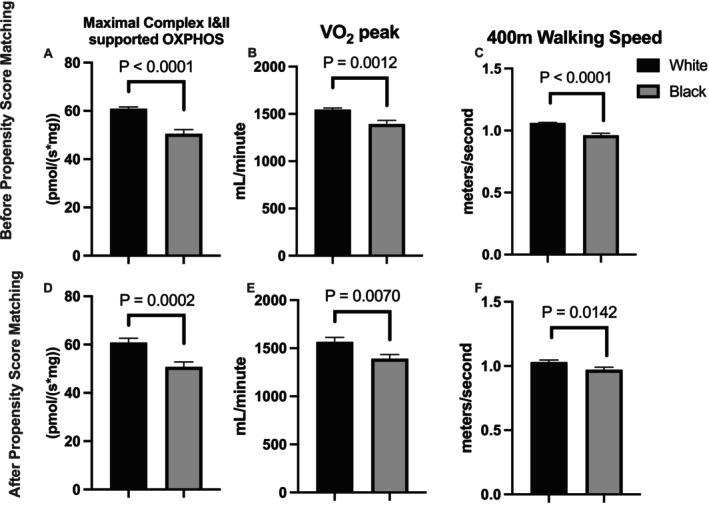
Race differences in mitochondrial respiration (Max OXPHOS), VO_2_ peak, and 400‐m walking speed. Group comparisons are made for the whole cohort before propensity score matching (White, *n* = 745 and Black, *n* = 116; Panels A–C), and in matched groups (White, *n* = 90 and Black, *n* = 90; Panels D–F) after propensity score matching. Panels A and D; Max OXPHOS in permeabilized fiber bundles from vastus lateralis biopsies. Panels B and E; Cardiorespiratory fitness (VO_2_ peak) from a cardiopulmonary exercise test (CPET) conducted on a treadmill using a modified Balke or manual protocol. Panels C and F; average walking speed during a 400‐m walk test conducted at the participant's usual or preferred pace for 10 laps around a 40‐m course. A two‐sample t‐test was used to test group differences. A value of *p* < 0.05 was considered statistically significant. Data are mean and standard error. Abbreviations: OXPHOS, oxidative phosphorylation.

**FIGURE 2:**
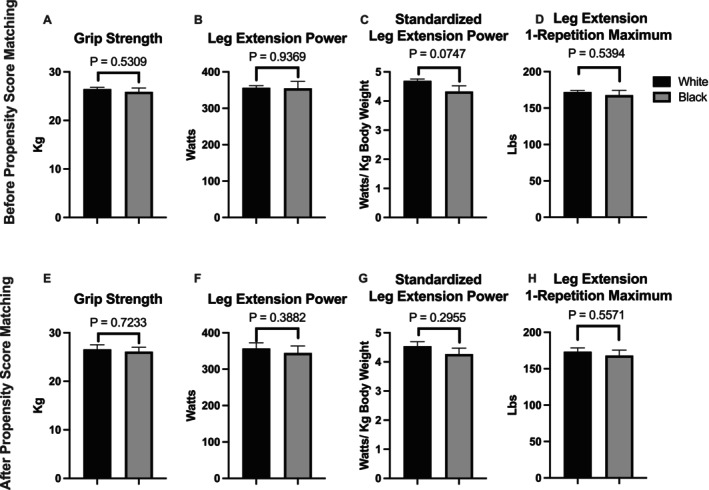
Race differences in grip strength, leg extension power, and strength. Group comparisons are made for the whole cohort before propensity score matching (White, *n* = 745 and Black, *n* = 116; Panels A–D), and in matched groups (White, *n* = 90 and Black, *n* = 90; Panels E–H) after propensity score matching. Panels A and E; Grip strength averaged between left and right hands using a Jamar hand‐held dynamometer. Panels B and F; Leg extension power using the Keiser AIR300 or A420 Leg Press system. Panels C and G; Standardized leg extension power (standardized to body mass). Panels D and H; Leg extension strength or one repetition maximum (1RM) was assessed using the Keiser AIR300 or A420 Leg Press system. A two‐sample t‐test was used to test group differences. A value of *p* < 0.05 was considered statistically significant. Data are mean and standard error.

Black participants also had slower 400‐m walking speed, lower muscle mitochondrial respiration (Maximal Complex I&II supported OXPHOS), and lower cardiorespiratory fitness (VO_2_ peak) (Figure 1, Panels A–C). In addition, there was no race difference in grip strength or leg extension power or strength (Figure 2, Panels A–D). To account for confounding variables that might bias group differences in walking speed, respiration, strength/power, and fitness, we used a propensity score matching approach to match Blacks with Whites in a 1:1 ratio. Socioeconomic variable selection for propensity score matching and subsequent models was based on variance explained in the outcome and group difference. Many variables, including mother/father education, having other investments, or adverse childhood events, did not explain variance in the outcome and so were not considered further. In addition, some variables also had a high level of missing data (participant income: 21.8%, current value of dwelling > $100,000: 75%) and were also not considered. Propensity scores were calculated by multiple logistic regression modeling using nine participant characteristics, including age, sex, SOMMA multimorbidity index, BMI, muscle mass, physical activity, marital status, educational achievement, and whether financial needs were met. The model selected groups of Black (*n* = 90) and White (*n* = 90) participants that were well matched for age, sex, BMI, D3Cr muscle mass, physical activity, and many other socioeconomic factors (Table 1 and Table S1). The results suggest that the propensity score matching improved model performance by reducing the confounding effects of the covariates and creating a more comparable sample. While financial variables were generally well matched, Black participants were less likely to have an IRA or KEOGH account, have other investments, or have stock or stock mutual funds. Black participants in the matched group were also less likely to report a value of their current dwelling of more than $100,000 (Table S1). Despite Black and White groups being well matched for physiological and socioeconomic variables, SOMMA participants who are Black had lower mitochondrial respiration (−16.6%, *p* < 0.001), VO_2_ peak (−11.2%, *p* = 0.007) and slower 400‐m walking speed (−5.8%, *p* = 0.014) when compared to the matched White participant group (Figure 1, Panels D–F). In addition, there remained no race difference in grip strength or leg extension power or strength (Figure 2, Panels E–H).

### 2.2 The Association Between Race and Slower 400‐m Walking Speed/Lower VO_2_ Peak Was Independent of Social and Economic Factors

We employed multivariate regression models using data from all participants and starting with a base model including sex, age, and clinical site as covariates. The base model indicated that Black participants had a significantly lower 400‐m walk speed compared to Whites (Table 2, β: −0.107 m/s, *p* < 0.001). In Model 2, the addition of marital status did not materially change the race difference (β: −0.101 m/s for Black vs. White race, *p* < 0.001). The inclusion of participant educational attainment, father job, and mother employment in Model 3 attenuated the difference, yet the association remained significant (β: −0.081 m/s, *p* < 0.001). For Model 4, financial need and stock ownership reduced the beta (group difference) by ~30%, but 400‐m walk speed remained significantly different between Blacks and Whites (β: −0.070 m/s, *p* = 0.001).

**TABLE 2:** Multivariable linear regression analysis for the association of race with 400 m and VO_2_ peak.

	Overall model				Group difference				
	*N*	*p* (model)	*r* ^2^	VIF	Lsmeans	Mean SE	Beta	Beta SE	*p*
**400‐m walk speed (m/s)**									
Base model: Sex, age, and clinical site	861	**< 0.001**	0.149	1.019	1.062	0.006	−0.107	0.016	**< 0.001**
Model 2: Base model + marital status	857	**< 0.001**	0.152	1.053	1.061	0.006	−0.101	0.017	**< 0.001**
Model 3: Base model + education, father job, mother employment	821	**< 0.001**	0.160	1.086	1.060	0.006	−0.081	0.018	**< 0.001**
Model 4: Base model + financial need, stock	780	**< 0.001**	0.181	1.116	1.062	0.006	−0.070	0.018	**< 0.001**
**VO_2_ peak (ml/min)**									
Base model: Sex, age, and site	803	**< 0.001**	0.576	1.018	1545.17	10.69	−145.58	31.50	**< 0.001**
Model 2: Base model + marital status	799	**< 0.001**	0.578	1.048	1545.88	10.70	−141.21	31.84	**< 0.001**
Model 3: Base model + education, father job, mother employment	770	**< 0.001**	0.583	1.078	1544.00	10.80	−116.64	33.59	**< 0.001**
Model 4: Base model + financial need, stock	731	**< 0.001**	0.598	1.101	1554.89	11.02	−90.78	35.19	**0.010**

*Note:* Boldface indicates significance accepted at *p* < 0.05.

A similar set of regression models was used to examine group differences in VO_2_ peak. The base model indicated that Black participants had a lower VO_2_ peak compared to Whites (Table 2, β: −0.145.58 mL/min, *p* < 0.001). In Models 2 and 3, the addition of marital status, participant educational attainment, father job, and mother employment attenuated the group difference in VO_2_ peak by ~20%, while the group difference remained significant (Beta *p* < 0.001). In Model 4, the addition of financial need and stock to the base variables reduced the beta (β: −90.78 mL/min) compared to the base model (β: −145.58 mL/min); however, the group difference remained statistically significant (*p* = 0.01).

### 2.3 The Association Between Race and VO_2_ Peak Is Not Explained by Muscle Mitochondrial Respiration (Max OXPHOS)

In Table 3, the base model corrected for sex, age, and clinical site shows that Black participants had a significantly lower VO_2_ peak compared to Whites (β: −145.58 mL/min, *p* < 0.001). Model 2 was built on the base model and included the following additional covariates: BMI, SOMMA comorbidity index, muscle mass, and step count. The results from Model 2 indicated that Black participants still had lower VO_2_ peak compared to White participants (β: −162.57 mL/min, *p* < 0.001). Finally, when Max OXPHOS was considered in Model 3, the beta value remained statistically significant, indicating that mitochondrial respiration did not explain the difference in VO_2_ peak between participants who are White and those who are Black (β: −137.21 mL/min, *p* < 0.001).

**TABLE 3:** Multivariable linear regression analysis for the association of race with 400 m and VO_2_ peak.

	Overall model				Group difference				
	*N*	*p* (model)	*r* ^2^	VIF	Lsmeans	Mean SE	Beta	Beta SE	*p*
**400‐m walk speed (m/s)**									
Base model: Sex, age, and clinical site	861	**< 0.001**	0.149	1.019	1.062	0.006	−0.107	0.016	**< 0.001**
Model 2: Base model + BMI, comorbidity, D3CR muscle mass, and step count	692	**< 0.001**	0.300	1.087	1.051	0.006	−0.068	0.018	**< 0.001**
sModel 3: Model 2 + VO_2_ peak	648	**< 0.001**	0.311	1.115	1.057	0.006	−0.041	0.020	**0.037**
Model 4: Model 2 + tech, Max OXPHOS	596	**< 0.001**	0.339	1.091	1.052	0.006	−0.037	0.019	0.056
Model 5: Model 2 + tech, Max OXPHOS, VO_2_ peak	561	**< 0.001**	0.354	1.110	1.058	0.006	−0.021	0.021	0.308
**VO_2_ peak (ml/min)**									
Base model: sex, age, and site	803	**< 0.001**	0.576	1.018	1545.17	10.69	−145.58	31.50	**< 0.001**
Model 2: base model + BMI comorbidity, D3CR muscle mass, and step count	648	**< 0.001**	0.694	1.071	1538.81	9.90	−162.57	31.75	**< 0.001**
Model 3: Model 2 + tech, Max OXPHOS	561	**< 0.001**	0.720	1.078	1553.47	10.32	−137.21	33.80	**< 0.001**

*Note:* Models including max OXPHOS are adjusted for technician. Boldface indicates significance accepted at *p* < 0.05.

### 2.4 The Association Between Race and Lower 400‐m Walking Speed Was Attenuated by Adjustment for Both VO_2_ Peak and Muscle Mitochondrial Respiration (Max OXPHOS)

Starting with a base model including sex, age, and clinical site as covariates, Black participants had a significantly lower 400‐m walk speed compared to Whites (Table 3, β: −0.107 m/s, *p* < 0.001). In Model 2, BMI, comorbidities, muscle mass, and step count were added, and while the beta was reduced, Black participants still had a significantly slower 400‐m walking speed compared to White participants (β: −0.068 m/s, *p* < 0.001). However, the addition of VO_2_ peak to Model 3 reduced the difference in 400‐m walking speed between Black and White participants (β: −0.041 m/s, *p* = 0.037). Similarly, the addition of Max OXPHOS in Model 4 also mitigated the group difference in walking speed (β: −0.037 m/s, *p* = 0.056). Finally, the addition of both Max OXPHOS and VO_2_ peak to Model 5 eliminated the difference in 400‐m walking speed between Black and White participants (β: −0.021 m/s, *p* = 0.308).

## 3 Discussion

Race disparities in health are a major economic, social, and healthcare issue in the United States. For example, older adults who identify as Black have a disproportionately higher incidence of mobility disability among other health conditions. We demonstrate in this analysis of the SOMMA cohort that slower walking speed in older adults who identify as Black compared to those who identify as White is explained by differences in muscle mitochondrial respiration and cardiorespiratory fitness. On the other hand, the association between race and slower walking speed was independent of SES factors and other physiological factors also known to influence walking speed and fitness. These findings are important as the relative contributions of SES factors, including education and income, along with physiological factors to explain race differences in mobility in a large cohort of older adults have not been adequately examined.

The observed race differences in walking speed in SOMMA are in line with other reports of lower physical functioning and mobility in older adults who are Black (Clark and Maddox 1992; Schoenbaum and Waidmann 1997; Thorpe Jr., Kasper, et al. 2008; Thorpe Jr. et al. 2011). In baseline data from the Health ABC study, which recruited only well‐functioning older adults, Black participants had slower walking speed than their White counterparts, a difference not fully explained by poverty, education, reading level, or income adequacy (Thorpe Jr. et al. 2011). Similarly, in the Women's Health and Aging Study, race and poverty‐related disparities in physical function were explored. Among the nonpoor, Black women had slower walking speed and reported more limitations in lower extremity function compared to nonpoor White participants, even after adjusting for demographic and health‐related variables (Thorpe Jr., Kasper, et al. 2008). Others have also demonstrated that race differences in measurable socioeconomic characteristics explain a substantial fraction, but not all, of Black/White differences in mobility and physical function (Guralnik et al. 1993; Schoenbaum and Waidmann 1997). Our data further these findings by demonstrating clear race differences in walking speed after controlling for SES (education, income, financial resources, marital status) and important physiological variables that significantly impact walking speed and that are different by race, including muscle mass, physical activity level, and BMI.

Several clinical trials and epidemiological studies have explored race differences in VO_2_ peak between Black and White participants. The vast majority of data indicate lower VO_2_ peak in Black compared to White participants (Arena et al. 2007; Carpenter et al. 1998; Hunter et al. 2000; Hunter et al. 2001). Data from Hunter et al. suggest that differences in hemoglobin and oxidative capacity of muscle partially explain reduced VO_2_ peak in Black women (Hunter et al. 2001). In our previous study, skeletal muscle characteristics, including mitochondrial density assessed by transmission electron microscopy, fat‐free mass, and maximal State 3 respiration, explained race differences in VO_2_ peak (Toledo et al. 2018). However, in SOMMA, muscle respiratory capacity did not explain race differences in VO_2_ peak. It is possible that other physiological factors that we have not measured also contribute to race differences in VO_2_ peak. In the Atherosclerosis Risk In Communities cohort study, Black race was associated with smaller left ventricular end‐diastolic dimension, higher relative wall thickness, and higher prevalence of concentric remodeling after adjusting for age, hypertension, BMI, diabetes, history of congenital heart disease, atrial fibrillation, and education level (Chandra et al. 2022). It may be that differences in cardiac structure and function also contribute to lower VO_2_ peak in older adults who are Black. Further studies are needed to more comprehensively assess the physiological contributors to lower cardiorespiratory fitness in Black individuals.

While others have shown that older Black adults have greater strength than older white adults and lower specific strength (Goodpaster et al. 2006; Newman et al. 2003), we did not see race differences in strength/power reflected in the SOMMA cohort. However, our results are in line with findings from the Baltimore Longitudinal Study of Aging, such that when SES is accounted for, body composition nor specific strength attenuated race differences in usual gait speed and fast 400‐m walk time (Chiles Shaffer et al. 2020).

The finding of lower muscle respiration in older adults who are Black is in line with a previous study from our group (DeLany et al. 2014), wherein we showed that Black women had lower muscle mitochondrial respiration and fitness compared to White women despite group matching for body composition and physical activity. Our results are also in line with data suggesting Black women have a lower oxidative capacity of muscle (Hunter et al. 2001) and a lower proportion of type I fibers (Ceaser and Hunter 2015; Tanner et al. 2002). In our previous study, lower succinate dehydrogenase staining, lower mitochondrial content assessed by electron microscopy, and lower mitochondrial respiration were observed in Black compared to White women. Significantly, lower mitochondrial respiration was still present after adjusting for either fiber type proportion or mitochondrial content, suggesting intrinsically lower mitochondrial respiration in individuals who are Black (DeLany et al. 2014).

Previous work from our laboratory and others revealed that mitochondrial function is a key property of skeletal muscle linked to VO_2_ peak and walking speed in older adults (Coen et al. 2013; Cummings et al. 2024; Gonzalez‐Freire et al. 2018). The well‐phenotyped SOMMA cohort provided an opportunity to assess the impact of VO_2_ peak and mitochondrial energetics on the association of race with walking speed while controlling for SES variables and objectively measured indices of physical activity, adiposity, muscle mass, and comorbidities. We report here for the first time that VO_2_ peak and mitochondrial respiration completely explain race differences in walking speed, but the race differences in walking speed are largely independent of SES variables and other physiological variables that did not explain a large proportion of the race difference in walking speed and VO_2_ peak. The implication of our work is that race disparities in mobility are likely more complex than previously thought, and further research is required to better understand the interrelationships between race, SES, and biological/physiological factors to better explain differences in mobility and other health disparities. Importantly, a life course approach needs to be considered, as a single assessment point in time may not capture a longer lifetime burden of worse health due to, for example, disparities in health resources. In addition, future clinical translational studies of well‐phenotyped and matched racial groups should focus on defining biological/physiological mechanisms responsible for race differences in mitochondrial respiration. Differences in fiber type proportion, oxidative stress/inflammation, mtDNA copy number, and integrity could all conceivably play a role. Studies of cardiac and vascular function would also provide valuable mechanistic insight into race differences in cardiorespiratory fitness.

There are some limitations to the study that should be considered. We acknowledge that our study participants were predominantly (85.6%) non‐Hispanic White, which limits our ability to relate our findings to other, more specific race/ethnic groups. Future studies with more diverse racial/ethnic cohorts are needed to further define differences in mobility with aging beyond comparisons of non‐Hispanic White versus Black participants. In SOMMA, all participants recruited were able to complete a 400‐m walking test, so our cohort had no major mobility issues. We had few participants who reported substantial financial insecurity or low income of either race; as such, economically impoverished populations are difficult to enroll in clinical studies. We did not have complete income and wealth measures for all participants. Therefore, we had an incomplete assessment of the broad nature of financial status that exists within the larger US population, and we may have found different results if such participants had been included. Nevertheless, we observe differences in physiological and functional aspects of skeletal muscle signifying the impact that self‐identified race has in this SOMMA cohort.

The strengths of our study are the large sample size of participants and rigorous assessment of objectively measured physical activity, fitness, and body composition, and collection of muscle biopsies for mitochondrial respiration assays. A strength of our analysis is that we considered a range of socioeconomic variables including education, income, financial resources, and marital status. This is important as self‐identified race and SES are highly associated, which makes examining race differences in mobility, fitness, and muscle phenotype per se more challenging. There are other SES factors that were not measured in SOMMA that might also contribute to apparent race differences in mobility. These factors may include historical discrimination and intergenerational transfer of wealth (Braveman et al. 2005; LaVeist 2005) or differences in debt burden and access to medical services (Bowen and Gonzalez 2008; Dunlop et al. 2002). Residential segregation is another possible factor to consider (Thorpe Jr., Brandon, and LaVeist 2008), as Blacks and Whites frequently live in different social and environmental contexts that might impact access to resources and opportunities that are important to maintaining mobility, for example, access to safe and hospitable walking routes. The implication is that there are potentially other social factors at play, ones that have not been measured or the ones we did measure in SOMMA maybe do not reflect a lifetime accumulation of adverse social factors, that in combination with genetic ancestry may explain race differences in VO_2_ peak and mitochondrial energetics. Further studies are needed to elucidate the complex relationships that contribute to race differences in mobility. In summary, while race differences in mobility are multifactorial and complex, our data indicate that muscle mitochondrial respiration and cardiorespiratory fitness may contribute to a slower walking speed of older individuals who identify as Black compared to White.

## 4 Experimental Procedures

### 4.1 Study Design and Participant Recruitment

SOMMA (https://www.sommaonline.ucsf.edu) is a longitudinal, observational multicenter study of aging (Cummings et al. 2023). Eight hundred and seventy‐nine older adults aged 70+ years were recruited between April 2019 and December 2021 from the University of Pittsburgh and Wake Forest University School of Medicine. Individuals were eligible to participate if willing and able to complete a percutaneous biopsy of the vastus lateralis and magnetic response spectroscopy (MRS) assessments. Individuals who reported active malignancy or advanced chronic disease had a BMI > 40 kg/m,^2^ and/or were unable to walk ¼ mile or climb a flight of stairs were excluded. The ability to complete a 400‐m walk within 15 min was determined at enrollment. The complete study design and assessment details have been described elsewhere (Cummings et al. 2023). All SOMMA participants provided written informed consent, and the study was approved by the Western Institutional Review Board (protocol #20180764). SOMMA data from May 2023 were used for the analysis.

### 4.2 General Study Measures

Participant baseline assessments were completed over several days and included self‐report standard questionnaires that captured sex, age, marital status, health history, medication record, diet, and physical activity. Race and ethnicity were based on self‐selection using categories required for reporting by the National Institutes of Health. For this analysis, we only considered participants who self‐selected Black/African American or White categories. No distinction was made based on self‐selected ethnicity. Weight was assessed by balance beam or digital scales and height by wall‐mounted stadiometers using standardized protocols.

### 4.3 Socioeconomic Status

Measures included household income (< $25,000; $25,000–50,000; $50,000–75,000; $75,000–125,000; > $125,000); housing arrangement (House; Apartment; Other); whether they own or rent (own; rent; other arrangement); if they own, the current value of the dwelling was recorded (> $100,000; > $175,000; > $350,000); perceived financial adequacy to take care of needs (categorized as poorly; fairly well; very well; don't know); financial adequacy to purchase food; whether the participant was receiving any free or subsidized food (food stamps; meals on wheels); financial adequacy at the end of each month (some money left; just enough money; not enough; don't know); Whether the participant (or partner) has savings or investments (checking/savings account; money market account; CDs, savings bonds or treasury bills; investment property; a business or farm; stock or mutual funds; an IRA or KEOGH account; other investments).

**Education level** of the participant was captured (no formal education; Grades 1–11; high school; some college; college graduate; postcollege; other; don't know). The education and occupation variables are from the Health and Retirement Study (HRS). Occupation is based on the 1988 Standard Occupational Classification (SOC) System. The employment history of the participant's father was recorded, as follows: Before age 16, was there a time of several months or more when your father had no job? (Yes; No; Father never worked or always disabled; Never lived with father or father was not alive). The employment history of the participant's mother was recorded, as follows: What portion of the time did your mother work outside the home when you were growing up? (None at all; Some of the time; All of the time; Never lived with mother/mother was not alive).

**Childhood adverse life events** of study participants were captured via a series of questions based on a modified version of the adverse childhood events (ACE‐Q) (Felitti et al. 1998). Six items were administered that assessed the number of adverse life events experienced during childhood (“While you were growing up (under 18) did you experience any of the following?”). Response items included (Yes; No; Don't know; and Prefer not to answer). A total of six items were summed to create a continuous measure (0–6).

### 4.4 SOMMA Multimorbidity Index

To construct the multimorbidity index in SOMMA, we curated a list of 11 age‐related chronic conditions that closely follow the Rochester Epidemiology Project (REP) (Espeland et al. 2017) and are described in more detail by Mau et al. (2024). The 11 conditions included cancer, CKD or renal failure, atrial fibrillation, lung disease (i.e., chronic obstructive pulmonary disease, bronchitis, asthma, or emphysema), coronary heart disease (i.e., blocked artery or myocardial infarction), depressive symptoms (e.g., ≥ 10 score on CESD‐10 consistent with depressive symptoms), heart failure, dementia, diabetes, stroke, and aortic stenosis. Participants were grouped into the number of chronic conditions they reported at the baseline visit, which was either 0, 1, or 2+ conditions.

### 4.5 Walk Speed Assessment

The 400‐m walk was conducted at the participant's usual or preferred pace for 10 laps around a 40‐m course without any assistive device other than a straight cane. The total time (seconds) to walk 400 m included the rest time if the participant stopped walking during the test.

### 4.6 Objectively Assessed Physical Activity

Participants wore the activPAL4 (PAL Technologies, Glasgow, Scotland, UK) accelerometer on their right thigh for seven consecutive 24‐h periods. Data were collected at 20 Hz, and proprietary algorithms used the accelerometer measurements to calculate the daily step count. The device was placed on the participants at the baseline Day 1 visit at the time of the 400‐m walk, and physical activity data were collected for a period of seven full days. The mean total daily step count was used as the index of daily physical activity. In addition, the Community Healthy Activities Model Program for Seniors (CHAMPS) questionnaire was used to calculate hours per week doing strength exercises and all walking activities (Stewart et al. 2001).

### 4.7 Whole Body Muscle Mass

Whole‐body D3Cr muscle mass was measured in participants using the d3‐creatine dilution method (Clark et al. 2014; Shankaran et al. 2018). Briefly, participants took a tablet with 30 mg of d3‐creatine and provided a fasting, morning urine sample 72–144 h later. The content of D3‐creatinine, unlabeled creatinine, and creatine in the urine was measured using high‐performance liquid chromatography and tandem mass spectroscopy (MS/MS). These data are then included in an algorithm to determine total body creatine pool size and skeletal muscle mass as previously described (Clark et al. 2014; Cummings et al. 2023; Shankaran et al. 2018).

### 4.8 Grip Strength and Leg Extension Power/Strength

Leg extension power and strength (one repetition max, 1RM) were assessed using the Keiser AIR300 or A420 Leg Press system. Grip strength was measured with a Jamar hand‐held dynamometer (Harkonen et al. 1993).

### 4.9 Cardiorespiratory Fitness (VO_2_ Peak)

Cardiorespiratory fitness was measured using the gold standard VO_2_ peak (mL/min) from Cardiopulmonary Exercise Testing (CPET). A standardized CPET, using a modified Balke or manual protocol, was administered to participants to measure ventilatory gases, oxygen, and carbon dioxide inhaled and exhaled during exercise (Balady et al. 2010; Wolf et al. 2024). Two slow 5‐min walking tests were conducted before and after the maximal effort test to assess walking energetics at a preferred walking speed and a slow fixed speed of 1.5 mph. Participants who were excluded from the maximal effort symptom‐limited peak test had acute electrocardiogram (ECG) abnormalities, uncontrolled blood pressure, or a history of myocardial infarction, unstable angina, or angioplasty in the preceding 6 months. Testing for VO_2_ peak began at the participant's preferred walking speed with an incremental rate (0.5 mph) and/or slope (2.5%) increased in 2‐min stages until the respiratory exchange ratio, the ratio between VCO_2_ and VO_2_, was ≥ 1.05 and the self‐reported Borg Rating of Perceived Exertion (Borg 1982) was ≥ 17. Blood pressure, pulse oximetry, and ECG were monitored throughout exercise. VO_2_ peak was determined in the BREEZESUITE software (MGC Diagnostics, St. Paul, MN) as the highest 30‐s average of VO_2_ (mL/min) achieved. The data were manually reviewed to ensure the correct VO_2_ peak was selected for each participant.

### 4.10 Skeletal Muscle Biopsy Collection and Processing

The biopsy was performed during a baseline clinic visit between 9 and 11 am while the participant was fasting (12 h) and had avoided strenuous physical activity for the prior 48 h. Percutaneous biopsies were collected from the middle region of the musculus vastus lateralis under local anesthesia using a Bergstrom cannula with suction. The specimen was blotted dry of blood and interstitial fluid and dissected free of any connective tissue and intermuscular fat. Approximately 20 mg of the biopsy specimen was placed into ice‐cold BIOPS media (10 mM Ca–EGTA buffer, 0.1 M free calcium, 20 mM imidazole, 20 mM taurine, 50 mM potassium 2‐[N‐morpholino]‐ethanesulfonic acid, 0.5 mM dithiothreitol, 6.56 mM MgCl2, 5.77 mM ATP, and 15 mM phosphocreatine [PCr], pH 7.1) for respirometry, as previously described (Mau et al. 2023). Myofiber bundles of approximately 2–3 mg were teased apart using a pair of sharp tweezers and a small Petri dish containing ice‐cold BIOPS media. After mechanical preparation, myofiber bundles were chemically permeabilized for 30 min with saponin (2 mL of 50 μg/mL saponin in ice‐cold BIOPS solution) placed on ice and on a rocker (25 rpm). Myofiber bundles were washed twice (10 min each) with ice‐cold MiR05 media (0.5 mM ethylenediaminetetraacetic acid, 3 mM MgCl2·6H2O, 60 mM K‐lactobionate, 20 mM taurine, 10 mM KH2PO4, 20 mM N‐2‐hydroxyethylpiperazine‐N′‐2‐ethanesulfonic acid, 110 mM sucrose, and 1 g/L bovine serum albumin, pH 7.1) on an orbital shaker (25 rpm). The second wash in MiR05 contained blebbistatin (25 μM), a myosin II ATPase inhibitor, that was used to inhibit muscle contraction. Fiber bundle wet weight was determined immediately after permeabilization using an analytical balance (Mettler Toledo, Columbus, OH).

### 4.11 High‐Resolution Respirometry

Myofiber bundles (2–3 mg) were prepared from the biopsy specimen by mechanical and chemical permeabilization for 30 min with saponin (2 mL of 50 μg/mL saponin in ice‐cold BIOPS solution). Mitochondrial respiration assays were performed using a standardized substrate uncoupler inhibitor titration (SUIT) protocol, which was run in duplicate to assess the activity of the mitochondrial electron transport system in permeabilized muscle fibers. The permeabilized muscle fiber bundles were weighed and transferred to the respiration chambers of an Oxygraph 2 K instrument (Oroboros Inc., Innsbruck, Austria). The assays were run at 37°C in MiR05 media supplemented with 25 μM of blebbistatin. The O_2_ concentrations were maintained between 400–200 μM within the respiratory chambers. Maximal complex I and II‐supported oxidative phosphorylation (Max OXPHOS/State 3 respiration) was measured in the presence of 5 mM pyruvate, 2 mM malate, 10 mM glutamate, 10 mM succinate, and 4.2 mM adenosine diphosphate (ADP). Cytochrome c (10 uM) was added to test the integrity of the outer mitochondrial membrane. When samples elicited an increase in respiration of > 15%, they were excluded from the analysis. Steady‐state O_2_ flux data were normalized to muscle fiber bundle wet weight using Datlab 7.4 software.

## 5 Statistical Analysis

### 5.1 Propensity Score Matching

Propensity scores were calculated by way of a multiple logistic regression model from nine characteristics of the participants. Those covariates were sex, age, BMI, current marital status, education level, whether financial needs were met, D3CR muscle mass, strength exercises and all walking (CHAMPS), and the SOMMA multimorbidity index. Matching for identical propensity scores resulted in two groups of 90 each out of a total of 861 participants with identical characteristics. In both the original study cohort and the propensity score–matched cohorts, differences between the two races were tested: continuous variables were tested using the two‐sample t‐test, and categorical variables were tested using Fisher's exact test. Continuous variables were presented using mean and standard error (SE) by race, and categorical variables were presented using frequency and proportion by race. A value of *p* < 0.05 was considered statistically significant. Statistical analysis was performed using SAS 9.4 (SAS Institute Inc., Cary, NC, USA).

### 5.2 Multilinear Regression

Linear regression was also used to determine the association of race with walking speed and VO_2_ peak. We evaluated the potential mediating influence of socioeconomic (Table 2) and physiological variables (Table 3). We did this by comparing the beta coefficient for the association of race on walking speed from the 400‐m walking test and VO_2_ peak. The base model included the confounding variables: sex, age, and clinical site. Subsequent models included different combinations of socioeconomic or physiological variables to further understand how these variables mediate the relationship between race and walking speed and VO_2_ peak. Those analyses were performed using SAS version 9.4 (SAS Institute Inc., Cary, NC).

## Author Contributions

Using Contributor Roles Taxonomy (CRediT), Paul M. Coen led the writing team for the original draft. Fanchao Yi and Li‐Yung Lui were co‐analysts who led formal analyses. Key conceptualization for this study involved Paul M. Coen and James P. DeLany. Li‐Yung Lui managed data curation and validation of the statistical analysis. Peggy M. Cawthon, James P. DeLany, Anne B. Newman, Steven R. Cummings, Stephen B. Kritchevsky, Bret H. Goodpaster, Russell T. Hepple, Gregory J. Tranah, Giovanna Distefano, and Sofhia V. Ramos provided the critical writing review and edits that led to the improvement of the manuscript. James P. DeLany and Paul M. Coen proofread multiple drafts of the manuscript. Steven R. Cummings, Peggy M. Cawthon, Anne B. Newman, Stephen B. Kritchevsky, and Russell T. Hepple enabled the study with either funding acquisition, project administration, supervision, and/or conceptualization for the study. All authors reviewed and approved this manuscript.

## Ethics Statement

The study protocol was approved by the Western Institutional Review Board Copernicus Group (WCG IRB; study number 20180764) and all participants provided written informed consent.

## Conflicts of Interest

Steven R. Cummings is a consultant to Bioage Labs. Peggy M. Cawthon is a consultant to and owns stock in MyoCorps. All other authors declare no conflicts of interest.

## Supporting information


**Table S1.** Race differences in additional socioeconomic characteristics. Group comparisons are made for the whole cohort before Propensity Score Matching (White, *n* = 745 and Black, *n* = 116), and in matched groups (White, *n* = 90 and Black, *n* = 90) after propensity score matching. Variables were tested using Fisher’s exact test and were presented using frequency and proportion by race. Boldface indicates significance accepted at *p* < 0.05.

## Data Availability

The data that support the findings of this study are available on request from the corresponding author. The data are not publicly available due to privacy or ethical restrictions.
