# Supplementary material for: Muscle Mitochondrial Respiration and Cardiorespiratory Fitness Contribute to Slower Walking Speed of Older Individuals Who Identify as Black
Source: Aging Cell. 2025 Mar 27;24(6):e70040. doi: 10.1111/acel.70040 (PMC12151876; doi:10.1111/acel.70040)
Supplement: Supplementary file 1 — Table S1. Race differences in additional socioeconomic characteristics. Group comparisons are made for the whole cohort before Propensity Score Matching (White, n = 745 and Black, n = 116), and in matched groups (White, n = 90 and Black, n = 90) after propensity score matching. Variables were tested using Fisher’s exact test and were presented using frequency and proportion by race. Boldface indicates significance accepted at p < 0.05. [file ACEL-24-e70040-s001.docx]

**Supplemental Table 1.**

|  |  | **Before PS Matching** | | | **After PS matching** | | |
| --- | --- | --- | --- | --- | --- | --- | --- |
| **Variable** |  | **White (n=745)** | **Black (n=116)** | **pvalue** | **White (n=90)** | **Black (n=90)** | **pvalue** |
| **Checking or savings account** | No | 3(0.4) | 2(1.9) | 0.125 | 0(0.0) | 2(2.3) | 0.497 |
|  | Yes | 713(99.6) | 103(98.1) |  | 88(100) | 86(97.7) |  |
| **A business or farm which you own** | No | 650(91.5) | 101(96.2) | 0.119 | 83(94.3) | 82(95.3) | 1.000 |
|  | Yes | 60(8.4) | 4(3.8) |  | 5(5.7) | 4(4.6) |  |
| **CDs, Saving bonds or treasury bills** | No | 433(63.4) | 79(79.0) | **0.002** | 68(77.3) | 65(79.3) | 0.853 |
|  | Yes | 250(36.6) | 21(21.0) |  | 20(22.7) | 17(20.7) |  |
| **Do you always have enough money to buy the food that you need** | No | 11(1.5) | 10(9.2) | **<0.001** | 5(5.6) | 9(10.0) | 0.405 |
|  | Yes | 720(98.5) | 99(90.8) |  | 85(94.4) | 81(90.0) |  |
| **Are you receiving any free or subsidized food, such as food stamps or Meals on Wheels** | No | 714(97.3) | 98(86.7) | **<0.001** | 84(93.3) | 76(85.4) | 0.095 |
|  | Yes | 20(2.7) | 15(13.3) |  | 6(6.7) | 13(14.6) |  |
| **Do you live in a house/apartment/other** | House | 635(85.8) | 88(78.6) | 0.117 | 72(80.0) | 68(75.6) | 0.688 |
|  | Apartment | 70(9.5) | 16(14.3) |  | 13(14.4) | 14(15.6) |  |
|  | Other | 35(4.7) | 8(7.1) |  | 5(5.6) | 8(8.9) |  |
| **Do you have an IRA or KEOGH account** | No | 157(22.9) | 70(70.0) | **<0.001** | 26(31.0) | 54(65.9) | **<0.001** |
|  | Yes | 528(77.1) | 30(30.0) |  | 58(69.0) | 28(34.1) |  |
| **Money market account** | No | 271(39.8) | 67(67.0) | **<0.001** | 49(57.0) | 52(63.4) | 0.433 |
|  | Yes | 410(60.2) | 33(33.0) |  | 37(43.0) | 30(36.6) |  |
| **In general, how do your finances usually work out at the end of the month** | Some money left over | 559(80.1) | 58(55.8) | **<0.001** | 61(69.3) | 50(58.1) | 0.229 |
|  | Just enough | 119(17.0) | 34(32.7) |  | 18(20.5) | 27(31.4) |  |
|  | Not enough | 20(2.87) | 12(11.5) |  | 9(10.2) | 9(10.5) |  |
| **Other investments** | No | 400(61.7) | 83(86.5) | **<0.001** | 58(69.0) | 68(86.1) | **0.014** |
|  | Yes | 248(38.3) | 13(13.5) |  | 26(31.0) | 11(13.9) |  |
| **Investment property or housing other than where you live** | No | 546(77.8) | 93(90.3) | **0.002** | 69(78.4) | 75(88.2) | 0.104 |
|  | Yes | 156(22.2) | 10(9.7) |  | 19(21.6) | 10(11.8) |  |
| **Do you own/rent/other arrangement** | Own | 655(88.8) | 80(73.4) | **<0.001** | 75(83.3) | 63(71.6) | 0.172 |
|  | Rent | 65(8.81) | 24(22.0) |  | 12(13.3) | 20(22.7) |  |
|  | Other arrangement | 18(2.4) | 5(4.6) |  | 3(3.3) | 5(5.7) |  |
| **Current value of dwelling - more than $100,000** | No | 37(20.1) | 19(59.4) | **<0.001** | 7(22.6) | 15(60.0) | **0.006** |
|  | Yes | 147(79.9) | 13(40.6) |  | 24(77.4) | 10(40.0) |  |
| **Current value of dwelling - more than $175,000** | No | 200(33.7) | 36(60.0) | **<0.001** | 34(50.7) | 29(59.2) | 0.451 |
|  | Yes | 394(66.3) | 24(40.0) |  | 33(49.3) | 20(40.8) |  |
| **Current value of dwelling - more than $350,000** | No | 202(58.9) | 19(90.5) | **0.004** | 18(72.0) | 15(88.2) | 0.271 |
|  | Yes | 141(41.1) | 2(9.5) |  | 7(28.0) | 2(11.8) |  |
